# Mobility Aware Duty Cycling Algorithm (MADCAL) A Dynamic Communication Threshold for Mobile Sink in Wireless Sensor Network †

**DOI:** 10.3390/s19224930

**Published:** 2019-11-12

**Authors:** Craig Thomson, Isam Wadhaj, Zhiyuan Tan, Ahmed Al-Dubai

**Affiliations:** School of Computing, Edinburgh Napier University, 10 Colinton Rd, Edinburgh EH10 5DT, UK; I.Wadhaj@napier.ac.uk (I.W.); Z.Tan@napier.ac.uk (Z.T.); A.Al-Dubai@napier.ac.uk (A.A.-D.)

**Keywords:** duty cycling, mobile sink, mobility awareness, wireless sensor network, dynamic threshold

## Abstract

The hotspot issue in wireless sensor networks, with nodes nearest the sink node losing energy fastest and degrading network lifetime, is a well-referenced problem. Mobile sink nodes have been proposed as a solution to this. They do not completely remove the hotspot problem though, with nodes the sink passes most closely still expending more energy than others. This study proposes a lightweight algorithm, located in the media access control (MAC) layer of static nodes and utilising knowledge of predictable sink node mobility. This is in order to create a dynamic communication threshold between static nodes and the sink, within which static nodes awaken, lessening competition for sink communication between nodes. In utilising predictable mobility and factors already known to the static node, such as location and interference range, there is no need for energy-consuming messaging. Analysis and simulation results, tested on a lightweight implementation of a carrier-sense multiple-access-based MAC protocol, show a significant improvement in energy consumption in both controlled and random environments, with frame delivery improved to the point where sink speed is negated. This is when compared to the existing duty cycling approach.

## 1. Introduction

Throughout industry and academia, wireless sensor networks (WSNs) have gained much attention in recent years. Comprising small, low power, limited-capacity devices, with data sent to a sink node on a many-to-one multi-hop basis. Given the environments in which these networks may be located, frequently inhospitable, with applications in many areas such as deep-sea oil and gas [1], disaster recovery [2], and agriculture [3,4], it may not always be possible to replace the batteries in network devices [5]. As a result, power conservation techniques which maximise node and, subsequently, network lifetime, take on importance. A common solution within WSNs to this issue is to duty cycle node power, such that when idle the nodes shall be asleep. However, whilst of benefit in terms of power consumption, duty cycling gives rise to other issues within WSNs. Heterogeneity of wake-up schedules within nodes in a WSN, as a result of duty cycling, results in problems with regard to neighbour discovery (ND), such that it becomes a challenge to ensure these schedules overlap in order that nodes may discover each other and data may be transmitted between them. This issue can then be exacerbated if mobility is also a factor within the network, ND schemes also now having to allow for nodes being within the same vicinity as well as an overlap of wake-up schedule.

This may occur when the sink node is mobilised in order to combat the particular issue of energy and routing hotspots [6] in static WSNs. These occur near the sink node as nodes closest to the sink will assume a greater responsibility in terms of routing than leaf nodes and, as a result, consume more energy. Ultimately, the lifetime of these hotspot nodes can be reduced considerably, with the other nodes in the network left unable to communicate with the sink node. Therefore, the WSN itself ceases to function properly. In utilising a mobile sink node (MSN), moving around or across the network, energy consumption is spread more evenly amongst the nodes in the WSN. This is due to no particular node being able to take on the role of hotspot for a considerable period of time. As a result, network lifetime can be increased and the hotspot issue negated to a certain degree [7,8]. The mobilising of sink nodes is possible via many different applications such as robots, vehicles, or being located on a person. However, the emergence of unmanned aerial vehicles (UAVs), commonly referred to as drones, provides further possibilities in this area, with studies having taken place to utilise drones in this way, to collect data and provide network connectivity [9]. As previously stated though, mobility adds another layer of complexity when considering ND and duty cycling. Sink mobility is no exception to this. Whereas in the use of MSNs, studies have taken place to extend existing work or, more specifically, develop new network layer routing protocols, thus far none have taken account of ND protocols. However, the issue of ND with regard to the mobility of devices in general in the internet of things (IoT), and specifically WSNs, has seen a great amount of research. With ND protocols in WSNs generally more concerned with duty cycling at the media access control (MAC) layer, referencing ND as a network layer issue has been consigned to older, more traditional fixed networks.

In networks consisting of small, battery-powered and duty cycled [10] devices, ND is no longer used to refer to IP addressing and duplicate address detection [11]. Now the greater concern is ensuring that ND is possible at all, with the overlap of wake-up schedules essential in this aim. In order to achieve this in an energy-efficient way, many probabilistic approaches have been proposed [12,13]. These techniques have been shown to be efficient, however, there is also the possibility that probabilistic methods may result in the log-tail discovery issue. This is where it may be the case that a node is not discovered at all [14]. As such, deterministic [15,16,17] algorithms occur more frequently in research. This is despite them being shown to be less efficient than probabilistic methods, but with the advantage of being able to guarantee an overlap, which probabilistic methods cannot claim. Recent studies have shown a move towards the adoption of new methods which may be integrated into ND. Opportunistic approaches involve decisions being made “on the fly” [18,19,20]. But it is in the area of mobility awareness in WSNs which is of interest within this study [21,22,23]. In this way it is proposed that routing and data delivery in a network may be improved by the prediction of mobile node mobility patterns [24]. However, in terms of influencing duty cycling and the wake-up schedule of nodes, as yet mobility has not been used in this area.

When considering the use of MSNs, many routing protocols have been developed for network layer solutions. These protocols can generally be separated into two categories, those being to implement flooding, which can result in high energy consumption, or delay-tolerant methods which compensate by being far more energy-efficient [8,25]. However, in more recent studies the use of MSNs has been combined with clustering and optimal sink path determination, such that more energy efficiency may be improved along with network layer routing [26]. Whereas these studies are valid when considering the potential difficulties in ensuring network layer packet delivery when an MSN is implemented in a WSN, it should be noted that it is at the MAC layer where most energy consumption can be found [22]. As such, it is proposed that it is at the MAC layer that any solution which aims to reduce energy consumption should be located, especially so when considering that this is where duty cycling takes place. Therefore, it is proposed that mobility patterns be used to influence duty cycling, such that nodes awaken at the most energy-efficient moment, when there is the greatest chance of any transmission reaching the sink node.

In this paper we propose a novel mobility-aware duty cycling algorithm (MADCAL) [27], to utilise the mobility pattern of the MSN in order to positively affect the wake-up schedules of static nodes. MADCAL operates at the MAC layer, independent of any routing protocol and uses no extra beacons or messages. The duty cycling of one-hop nodes to the sink is based on the current location of the MSN and a dynamic communication threshold, calculated within each node, independently of all others. This calculation uses the interference range of the node, its distance from the path of the MSN, and the speed of the MSN. When subsequently compared to standard duty cycling with clear channel assessment (CCA) and check interval, we demonstrate that MADCAL shows improvement in energy consumption across the network as well as in the number of frames received by the MSN.

This study extends our conference paper [27] as follows:

- Methodology. An adaptive communication threshold has been proposed in this new version to provide self-adaption to the changes of other network parameters.
- Algorithm. Both algorithms for the determination of a communication threshold are now described line by line in detail. Improvements, including new functionality, have been added to and highlighted in the second part of the MADCAL algorithm.
- Test scenarios. In this journal version we utilise both a random topology and the one grid topology involved in the conference paper to demonstrate the effectiveness of MADCAL even when the topology is not controlled. As such a more comprehensive evaluation is now delivered and test results are doubled in comparison with that of the conference paper. In addition, related work has been reviewed and classified in Section 2.

This paper is organised as follows. In Section 2 we discuss related work pertaining to this study. Section 3 describes our approach to mobility-aware duty cycling and the network scenarios utilised. In Section 4 the MADCAL algorithm is discussed in detail along with an illustrated description of how the communication threshold is calculated. Section 5 describes the simulation parameters and all test results along with the discussion. Finally, Section 6 contains a conclusion and our plans for future work.

## 2. Related Work

In reviewing related work, we highlight studies where sink mobility is utilised in order to affect various aspects of network performance. As such, we do not review particular routing protocols designed for use with sink mobility.

### 2.1. Network Improvements with Sink Mobility

In examining the most compelling reason for mobilising sink nodes, that being the improvement in energy consumption versus the use of a static sink node, [28] uses a linear mobility model and claims a 500% increase in network lifetime versus a static network. This operates by utilising a sojourn time, which is commonly referenced in other work, where the sink lingers at a node, in this case, to limit time until the first node runs out of power. More recent studies have progressed to novel approaches such as to strategically alter the sink trajectory, such as in [29] where trajectory is altered based on node density, with the aim to pass every single node. This utilises a novel approach of a “space-filling curve” [30]. This study suggests a 20% increase in packet delivery ratio (PDR) when a dynamic curve approach is in use. However, given the reliance on node density as a factor, scalability could become problematic. Energy consumption does not appear to be a priority within this study; also, at the MAC layer, 802.11 is used as standard, with no reference to this beyond test parameters. As such, whilst the results produced in this study have merit, the approach is not directly relatable to an MAC layer approach to sink mobility.

Sink trajectory is again a factor in [31]. A real-world application of an MSN, the FarmBeats platform is developed for precision architecture by using algorithms developed to ensure effective path-planning to extend the battery life of drones—the MSNs in this scenario. This paper also makes reference to the importance of duty cycling in the base stations but does not base this on the mobility of the MSN. This work is extended in [32] by additionally utilising two residual energy thresholds, one for the entire network and one for the path of the MSN. This is then used to modify the existing FarmBeats [31] algorithms in terms of duty cycling and path selection. The authors claim benefits in terms of processing time and energy performance.

### 2.2. Mobile Sink Node Optimal Path

Optimal path planning is in evidence in [6]. One of the main features of this paper is a secondary approach to prioritise emergency broadcasts. One observation to be made in this paper is that the static nodes shall awaken when the sink node is nearby. However, how this happens is not detailed and would appear to be one of the assumptions on which the subsequent work in the study is built. This study demonstrates the benefit of utilising sink mobility to influence network behaviour and results demonstrate improvement in network lifetime and transmission delay. The approach here is to adjust the mobility pattern of the MSN to coincide with the duty cycling algorithm in use. The benefits of this approach are proven as are the potential advantages in linking sink mobility to duty cycling.

Pazzi et al. [33] propose the eTrail protocol. In this study, the MSN acts as the cluster head, with clusters built as the sink moves. The MSN sends beacons in order to leave a trail of the sink’s path, with sensor nodes updating their routing information so that the path to the sink is kept up-to-date for each node. The authors claim minimal communication overhead due to the use of only local broadcasts, with this work located at the network layer. The use of beacons and broadcasts of any kind is something we have looked to eliminate within our own studies given the increase in energy expenditure that comes with their use.

### 2.3. Mobile Sink Nodes and Delay

The primary focus of [34] is to address the issue of delay when implementing a MSN. In particular, when considering delay-sensitive data which should not be subject to the same restrictions placed upon other data. In this case the authors developed a delay-intolerant routing scheme (DRS), implementing a deadline by which data should be received by the sink. When a sensor has emergency data to transmit it will either wait until the sink is within its grid or send immediately to an awake sensor node. Again, this is a study which takes place at the network layer, with regard to the routing scheme implemented. However, reference is made to the probable effectiveness of controlling the wake-up pattern of nodes alongside this scheme.

## 3. Mobility-Aware Duty Cycling

### 3.1. Beacon Messaging

The related work we have reviewed demonstrates a propensity in studies to determine, by various different parameters, an optimal path for the MSN, which has merit in the results of each particular work. However, a common theme to be found is in the approach taken to keep track of the MSN. The regular exchange of beacon messages can have a significant negative effect on the energy consumption of a network. Our new mobility-aware duty cycling approach utilises a pre-defined mobility pattern. Our hypothesis is that given network parameters such as sink start position, speed, and time, each static node is capable of independently calculating the current sink position. This can therefore be achieved without the expensive exchange of messages, with the result being a lightweight algorithm with no network overhead.

### 3.2. Significant Nodes

The first step in this study is to identify the significant nodes with which the MSN shall communicate directly as it travels along its path. These nodes now take on the role of hotspot, with responsibility for communication with the sink node spread amongst them. We aim to reduce energy consumption amongst these significant nodes the MSN communicates with via one-hop, while also improving the number of frames delivered to the sink node, or at least keeping that figure within a reasonable boundary.

### 3.3. Mobility Pattern

When considering the mobility pattern used, we seek to study an environment where all nodes are not treated equally, such as a disaster recovery situation where reaching each node directly is not possible. Many studies aim to have the MSN pass every node and whilst such a mobility pattern has merit, it is clear that this is not always possible in a real-world scenario. Therefore, in our tests we have implemented a circular mobility pattern for the MSN, moving around the periphery of the network.

### 3.4. Network Topology

**Figure 1:**
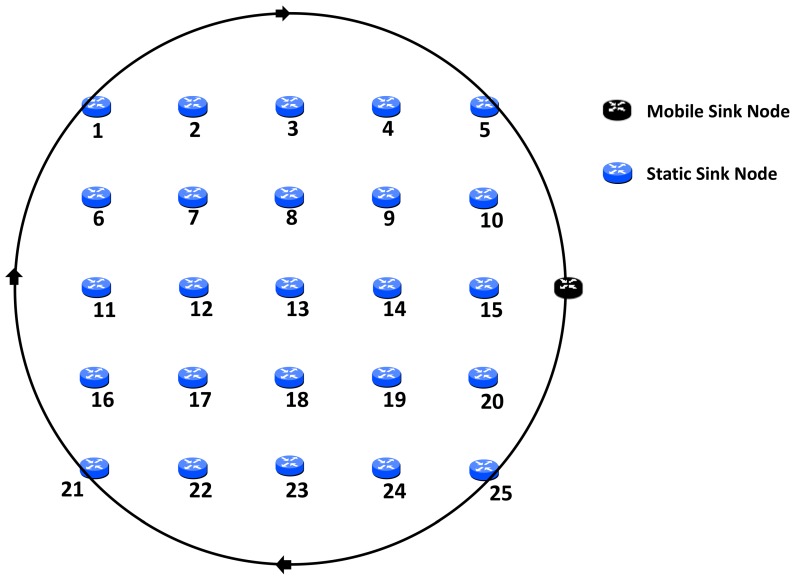
Network topology—grid.

With regard to network topology, we have taken two approaches. Firstly, a one-hop grid formation is used. This in order that results could be observed when controlled location of static nodes was in effect. As such, it is easier to observe the effect that communication with the MSN has on the static nodes in the network, with both interference range and distance from the path of the sink node reasonably consistent. Figure 1 shows the network layout, with the start point of the MSN and the clockwise direction of travel.

**Figure 2:**
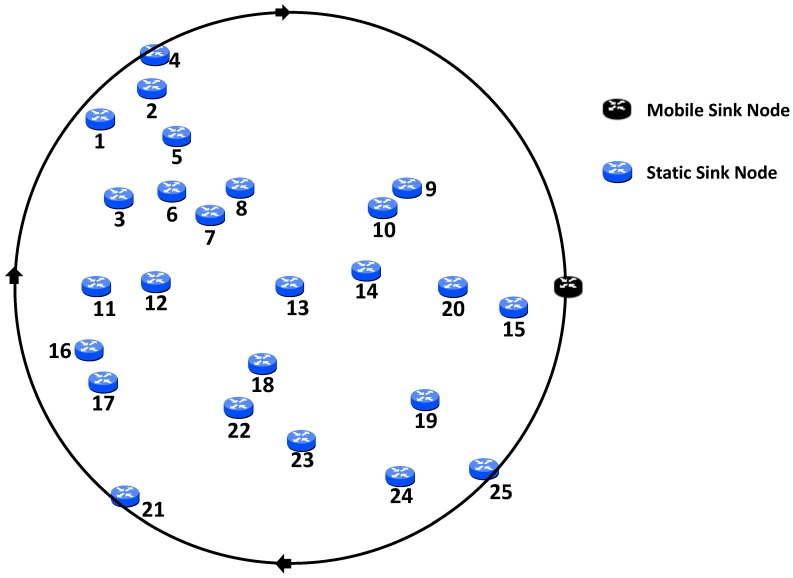
Network topology—random.

In addition, a more random topology has been deployed in order to ensure that MADCAL will also work in more diverse circumstances. This can be seen in Figure 2.

As can be observed, depending on the interference range, certain nodes within the network will be within one-hop of the MSN. These are determined to be the aforementioned significant nodes, taking the place of hotspot nodes in a network where the sink node remains static, only now the significant nodes take it in turn to have the final responsibility for relaying data to the sink. As the ultimate aim is to reduce energy consumption and increase network lifetime, this benefit is negated if only certain nodes have channel access and thus, the ability to communicate with the sink node. Whilst other nodes wait for the channel to become clear before communication with the sink can commence. Therefore, we propose the development of a threshold of communication in order to ensure fair access to the MSN.

### 3.5. MAC Implementation

**Figure 3:**
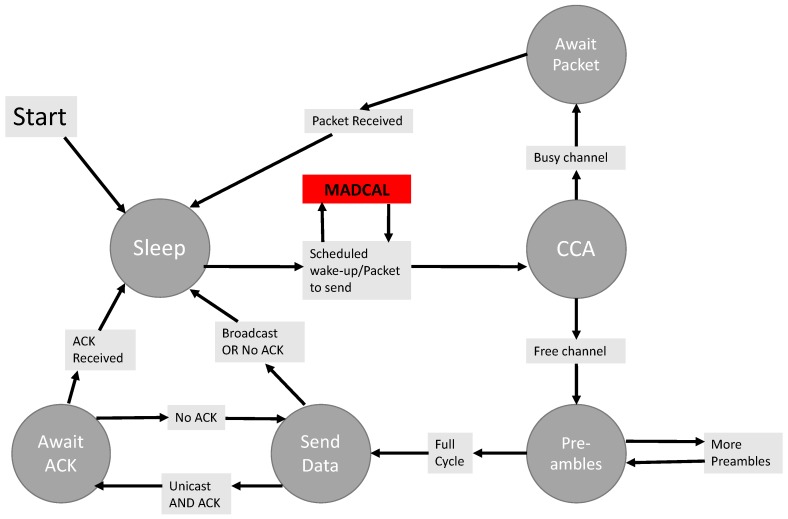
Media access control (MAC) implementation.

The MADCAL algorithm is implemented at the MAC layer, where the greatest amount of energy is consumed amongst network layers [35]. The MAC implementation utilised in this study is a lightweight carrier-sense multiple access (CSMA) implementation which reflects the core functionality of the IEEE 802.15.4 standard [36,37]. This uses CCA and the transmission of preambles. Figure 3 demonstrates this MAC implementation, with the location of the MADCAL functionality highlighted to show how the normal wake-up schedule is intercepted.

### 3.6. Network Properties

The following properties of the WSN are assumed:

1. Static node positions are constant throughout.
2. Static nodes are aware of their own location.
3. Static nodes are unaware of the location of neighbouring nodes, each node implements the MADCAL algorithm independently.
4. Node power levels are consistent.
5. Interference ranges, though variable across tests, are consistent across static nodes and the MSN.
6. Sink speed shall not be less than 2 mps or greater than 40 mps.

### 3.7. Simulation Parameters

In reference to the simulation parameters in Table 1, the simulation time is calculated to ensure an exact number of circuits of the network by the MSN. As such, with the speed at 2 mps (meters per second) the sink shall complete exactly 2 circuits of the network, for 10 mps, 10 circuits and so on.

Interference distance is calculated as thus [38]:

(1) $$interferenceDistance={\frac{{\left(\frac{SoL}{Freq}\right)}^{2}*Power}{\left(16*PI^{2}*10^{\frac{SAT}{10}}\right)}}^{\frac{1.0}{Alpha}},$$

where *SoL* denotes the speed of light (i.e., 30,000,000 mps); *Freq* stands for the carrier frequency; *Power* indicates the transmitter power; *SAT* is the signal attenuation threshold; and *Alpha* represents the path loss alpha.

Received signals with power below the sensitivity value are ignored. In this case the value was adjusted from −85 dBm to −75 dBm in order to reduce the number of signals received and thus lessen the risk of network failure due to node overload.

All parameters are consistent across all simulation runs apart from the speed of the sink node and the interference distance of the nodes. The path loss alpha is adjusted across four different values, as detailed in the test parameters; this in order to alter the size of the interference distance, which decreases as the alpha value increases.

### 3.8. Network Layer

This study does not seek to test routing protocols. However, a routing protocol is required in order to ensure final delivery is to the MSN. Otherwise, MAC layer frames would simply be sent in bursts, with the MSN behaving as any other node in the network would. Given this, the optimized link-state routing protocol [39] (OLSR) is utilised in this study. This is an unconventional approach as OLSR is not usually used in a WSN environment. However. OLSR is a resource-heavy protocol which places a great load on the network, especially in terms of energy consumption. For our purposes this proved to be of benefit and resulted in accelerated tests, requiring a lower simulation time in order to acquire the desired results.

## 4. Mobility Aware Duty Cycling Algorithm (MADCAL)

### 4.1. The Communication Threshold

The basic premise of MADCAL is to establish a threshold of communication between each significant static node and the MSN, with significance based on whether the distance from the static node to the path of the sink is less than the node’s interference range. In the case of a circular sink mobility pattern as utilised here, this involves establishing the coordinates of the start and end of the portion of the circle circumference where, when reached by the MSN, the particular static node should be awake for communication. This threshold is calculated during the initialisation stage of each static node and is detailed in Algorithm 1.

**Algorithm 1** Communication threshold.
1:
**procedure** initialisation
2:    set sinkSpeed3:    significantNode←false4:    set interDist5:    set Circumference6:    set firstSinkPos7:    set firstSinkQuartile8:    set distToCircle9:    **if** distToCircle<interDist **then**10:        significantNode←true11:    **end if**12:    **if** significantNode **then**13:        set circlePoint14:        set nodeQuartile15:        set distanceBetweenPoints16:        set angleOfNode17:        thresholdAfter←true18:        sinkThresholdAfter←establishThreshold(sinkRadius,thresholdAfter)19:        thresholdAfter←false20:        sinkThresholdBefore←establishThreshold(sinkRadius,thresholdAfter)21:        set thresholdDistance22:        set beforeQuartile23:        set thresholdOpposite24:    **end if**25:
**end procedure**
26:**function**establishThreshold(radius, after)27:    nodeDist←(radius-distToCircle)28:    $angleTemp\leftarrow \frac{\left(radius^{2}+nodeDist^{2}-interDist^{2}\right)}{\left(2*radius*nodeDist\right)}$29:    angleRadians←arccos(angleTemp)30:    $angle\leftarrow (angleRadians*\left(\frac{180}{PI}\right)$31:    $factor\leftarrow \frac{distToCircle}{interDist}$32:    **if** sinkSpeed<10 **then**33:        factorCheck←0.534:    **else if** sinkSpeed<20 **then**35:        factorCheck←0.3536:    **else if** sinkSpeed<40 **then**37:        factorCheck←0.2538:    **end if**39:    **if** factor<factorCheck **then**40:        factor←factorCheck41:    **end if**42:    angle←(angle∗factor)43:    **if** after **then**44:        threshAngleDegrees←(angle+angleOfNode)45:    **else**46:        threshAngleDegrees←(angleOfNode-angle)47:    **end if**48:    $threshAngleRadians\leftarrow \frac{threshAngleDegrees}{\left(180*PI\right)}$49:    threshold.x←circleCentre.x+(radius∗cos(threshAngleRadians))50:    threshold.y←circleCentre.y+(radius∗sin(threshAngleRadians))51:    **return** Coord threshold52:
**end function**

- Lines 1–3—initialisation. The MSN speed is set from input (2, 10, 20, or 40 mps), significant node not established yet.
- Line 4—the interference distance, interDist, of the node is calculated as per the algorithm previously stated.
- Lines 5–7—the circumference of the circular path of the MSN is calculated, with the coordinates of the start point of the MSN set from input as firstSinkPos. Based on the sink start point, the quartile of the circle the sink initially resides in is calculated as firstSinkQuartile. These quartiles can be described as north–west, north–east, south–west, or south–east.
- Line 8—the shortest distance from the node to the circular path of the MSN is calculated as distToCircle.
- Lines 9–11—if the distance to the circular path is less than the node interference distance, then the node is deemed to be significant, in that it shall be able to communicate directly with the MSN at some point.
- Lines 12–14—coding relevant to significant nodes. The coordinates of the closest point to the circular path from the node are calculated as circlePoint, and the quartile in which the circlePoint resides calculated as nodeQuartile.
- Line 15—the distance between firstSinkPos and circlePoint in a straight line is calculated as distanceBetweenPoints.
- Line 16—calculate the angle of the circlePoint as angleOfNode, between 0 and 360 degrees, with zero the farthest east point of the circle and default starting point for the sink, although a zero sink start point is not compulsory. Calculation based initially on the firstSinkQuartile and nodeQuartile in order to ascertain the angle between sink start point and circlePoint.
- Lines 17, 18—thresholdAfter is set to true in order that the coordinates of the threshold after the circlePoint may be calculated. The coordinates of the threshold after the circlePoint are calculated as sinkThresholdAfter using the establishThreshold function.
- Lines 19, 20—thresholdAfter is set to false in order that the coordinates of the threshold before the circlePoint may be calculated. The coordinates of the threshold before the circlePoint are calculated as sinkThresholdBefore using the establishThreshold function.
- Line 21—calculate the distance in a straight line between the two threshold coordinates, before and after, as thresholdDistance.
- Lines 22, 23—calculate the quartile in which sinkThresholdBefore is located as beforeQuartile, followed by the coordinates of the opposite point to the threshold as thresholdOpposite, for use later in determining the sink position in relation to the node.
- Lines 24, 25—the end of both coding only relevant to significant nodes and the initialisation procedure.
- Line 26—start of the function to establish the coordinates of the communication threshold, inputting the radius of the circular path and whether the coordinates to be calculated are before or after the circlePoint. The threshold point is based upon a combination of node location and the point at which communication with the sink should no longer be possible, based on interference distance.
- Line 27—calculate the distance from the centre of the circle to the static node as nodeDist.
- Lines 28–30—calculate the angle of the circle centre to the furthest point of communication, and the circle centre to the node.
- Line 31—in order to avoid excessively large thresholds, determine a factor by which this angle should be reduced based upon distToCircle divided by interDist.
- Lines 32–38—establish a factor check based upon the speed of the MSN. The faster the sink speed may be, the more the angle of the threshold may be reduced by.
- Lines 39–41—if the factor calculated is less than the factor check then the factor check value becomes the factor.
- Line 42—the value of the angle of the threshold is multiplied by the factor in order to reduce it accordingly.
- Lines 43–48—if sinkThresholdAfter is being calculated then the angle calculated is added to angleOfNode, otherwise the angle is subtracted from angleOfNode. This results in threshAngleDegrees. The threshAngleDegrees is then converted to radians.
- Lines 49–52—the x and y coordinates of the threshold are calculated, returning the coordinate value threshold. The establishThreshold function ends.

### 4.2. Initial Calculation of Threshold

**Figure 4:**
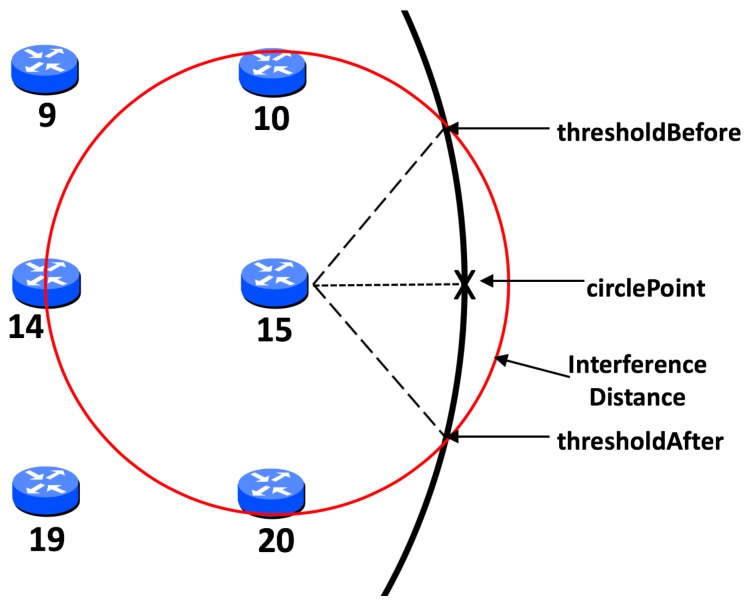
Illustration of initial threshold calculation.

With circular mobility, this threshold is calculated based on the angle of the closest point to the circular path in relation to the static node—the circlePoint. Taking into account the interference range of the node and the radius of the circle, an initial maximum threshold before and after the circlePoint can be calculated. This is demonstrated in Figure 4 in relation to node 15. However, a simplistic approach such as this would result in a significantly large threshold if the static node is close to the path of the MSN. In this event, this node could monopolise communication with the sink for a considerable time, to the detriment of other significant nodes. To negate this, a more dynamic approach to calculating the threshold is required. Firstly, node distance to the sink path is taken into consideration and divided by the interference distance, in order to create a factor by which the threshold angle shall be multiplied. Secondly, in order to now avoid an extremely small threshold for nodes closer to the path, a factor check is utilised. This lessens how much the threshold is reduced based upon the speed of the sink node, which is constant throughout each scenario. It was found, via simulation, that as the sink speed increases a smaller threshold is more efficient, given that the sink shall pass through this threshold more often. For example, in our experiments, if the sink node is travelling at 40 mps it shall pass through the threshold of any significant node 40 times in each scenario, giving each significant node many opportunities to communicate with the sink. However, if the sink node is travelling at just 2 mps it shall only pass through the threshold of each significant node two times in each scenario. In this case, it would make sense for thresholds to be larger to give each significant node as much chance as possible to communicate with the sink.

Therefore, as a first approach, the factor check is utilised such that for speeds less than 10 mps the threshold cannot be reduced by less than a factor of 0.5, for less than 20 mps this factor reduces to 0.35, reducing again to no less than 0.25 for less than 40 mps. If the speed is exactly 40 mps we take no action to use a factor check, allowing the threshold to be reduced based upon the initial calculation of node distance over interference distance. It is envisaged that in future work an extension to this algorithm could be developed to ensure the factor is completely dynamic based on the speed of the sink node.

### 4.3. Threshold Calculation with Factoring Employed

Using node 15 again as an example, which has a distance of exactly 50 m from the path of the MSN. In the example shown in Figure 4 the interference range is 77.52 m which would give an initial factor calculated as thus:

(2) $$factor=\frac{distToCircle}{interDist},$$

which in this case would result in:

(3) $$\frac{50}{77.52}=0.645.$$

**Figure 5:**
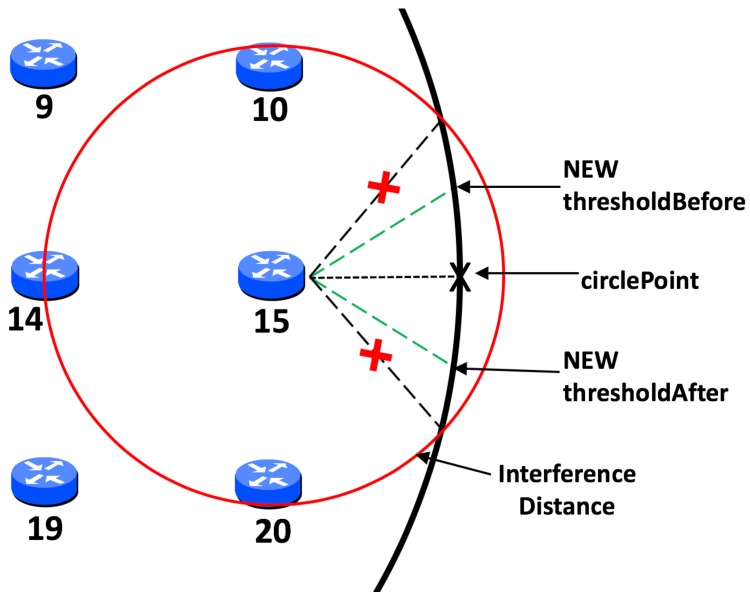
Adjusted threshold calculation.

Assuming a speed of 2 mps then speed is less than 10 mps, therefore the factor check is set to 0.5. This would be compared against the result of the initial calculation which, if less than the factor check, would result in the factor check value, 0.5, being assigned as the value of the factor, ensuring it can not be reduced by less than 0.5. However, in this case this is not necessary and the angle of the threshold would be reduced by a factor of 0.645 as shown in Figure 5.

### 4.4. Duty Cycling Adjusted with MADCAL

Algorithm 2 is designed to be inserted within the existing MAC code in order to establish the node wake-up time. This is based on calculating the current sink position by utilising the sink start position, the size of the circle circumference, and the current simulation time. This enables the static nodes to calculate the sink position without the need for beacons or other energy consuming methods such as a global positioning system (GPS) [40]. The sink position is then compared to the coordinates of the start of the threshold calculated in Algorithm 1, with node wake-up time determined by how long it will take the sink to reach the threshold, or else reverting to the original code using the check interval as input, in the event the sink is already within the threshold.

**Algorithm 2** Threshold interval.
1:
**procedure** sleep
2:    set checkInterval3:    **if** significantNode **then**4:        thresholdTime()5:        **if** thresholdReached **then**6:           interval←checkInterval7:        **else**8:           interval←timeToThreshold9:        **end if**10:    **else**11:        interval←checkInterval12:    **end if**13:    schedule WAKEUP at simTime + interval14:
**end procedure**
15:
**function** thresholdTime
16:    set sinkPos17:    withinThreshold()18:    **if** not thresholdreached **then**19:        set arc to distance to sinkThresholdBefore20:        set sinkQuartile21:        $timeToThreshold\leftarrow \frac{arc}{sinkSpeed}$22:    **else**23:        timeToThreshold←024:    **end if**25:    **return** timeToThreshold26:
**end function**
27:
**function** withinThreshold
28:    **if** distance between sinkPos and thresholdAfter > thresholdDistance **then**29:        thresholdReached←false30:    **else if** distance between sinkPos and thresholdBefore > thresholdDistance **then**31:        thresholdReached←false32:    **else**33:        thresholdReached←true34:    **end if**35:    **return** thresholdReached36:
**end function**

- Lines 1, 2—the Sleep procedure begins. The default sleep interval is set as checkInterval.
- Lines 3–9—applies to significant nodes only. The thresholdTime function is called to establish the time it will take for the sink to reach the threshold. If the threshold has been reached, the interval to wake-up reverts to the checkInterval; otherwise, it is set to the time it will take for the sink to reach the threshold.
- Lines 10–12—if this is not a significant node the wake-up schedule reverts to the checkInterval.
- Lines 13, 14—the wake-up is calculated by adding the interval to the current simulation time, the Sleep procedure ends.
- Line 15—the start of the thresholdTime function to establish how long it will take the sink to reach the threshold.
- Lines 16, 17—the current sink position is calculated as sinkPos based on simulation time and the initial sink start position, with the withinThreshold function called to establish if the sink is already within the communication threshold or not.
- Lines 18–21—if the threshold has not been reached yet, set the arc to the distance the sink must travel to reach the sinkThresholdBefore coordinates. Establish the current quartile in which the sink resides and calculate the time left to reach the threshold as the size of the arc in metres divided by the speed of the MSN in mps.
- Lines 22–24—the threshold has been reached, therefore the time left to reach the threshold is set as zero.
- Lines 25, 26—the timeToThreshold is returned and the thresholdTime function ends.
- Line 27—the start of the withinThreshold function to establish the position of the sink node in relation to the static node communication threshold.
- Lines 28, 29—establish if the distance between the current sink position and the thresholdAfter coordinates is greater than the size of the entire threshold. If so then the threshold has not been reached and thresholdReached is set to false.
- Lines 30, 31—however, if this is not true and the distance between the current sink position and the thresholdAfter coordinates is less than the size of the entire threshold. Now establish if the sink is before or after the threshold. If the distance between the sink position and the thresholdBefore coordinates is greater than the thresholdDistance then the sink must be beyond the threshold and therefore thresholdReached is set to false.
- Lines 32–34—if the sink is also within thresholdDistance of thresholdBefore however, this means the sink is within the threshold. Therefore thresholdReached is set to true.
- Lines 35, 36—thresholdReached is returned and the withinThreshold function ends.

## 5. Evaluation and Results

### 5.1. Simulation Environment and Parameters

Work was conducted on the OMNeT++ [41] framework, a platform on which simulations can be built. As such, in this study MiXiM [42] was utilised to build the network environment, including the location of nodes and the size of the actual area within which they are located. In addition, inetmanet [43] was used for all other factors, including physical, MAC, network, and transport layer parameters, as well as the use of mobility and energy models.

### 5.2. Energy Model

The energy module was the commonly used InetSimpleBattery module, found within inetmanet [43]. This module added little to overhead in terms of computation with a lightweight estimation of energy consumption. As such, this was utilised by the physical layer to receive energy level values in order to facilitate operation of the wireless adaptor [44,45,46].

### 5.3. Test Scenarios and Results

Each scenario utilised one of four different interference ranges, consistent across all nodes and the sink. This demonstrated the effectiveness of the MADCAL algorithm as interference ranges began generously and were then contracted, to the point where they barely covered one-hop between nodes. Within each scenario the MSN speed was altered between 2 mps, 10 mps, 20 mps, and 40 mps. Therefore, results can be compared between the sink moving very slowly, only encountering individual nodes a small amount of times, to a high sink speed meaning that many passes of each node were possible in the same simulation time.

Results were first obtained for the network implementation with an MSN, but with the existing standard duty cycling with CCA and check interval. This made no allowances for sink mobility. Result metrics were of average energy consumption amongst significant nodes and MAC layer frames received by the sink node. Tests were conducted using both topologies as illustrated in Figure 1 and Figure 2. The most significant difference across the two topologies was in the assignment of significant nodes. Node location was more stable and, although interference range was altered across tests, the significant nodes did not change and remain as nodes 1–6, 10, 11, 15, 16, and 20–25. However, in the case of the random topology, as interference ranges were altered, so were the significant nodes. This is covered in greater detail later.

### 5.4. Grid Network Formation

#### 5.4.1. Static Network

As a reference point, tests were conducted with the same simulation time but with the sink node immobile and remaining at the start position of the MSN, next to node 15, as shown in Figure 1. What was found was unless there was a large interference distance which could encompass more than one node, this one node used up the most energy. However, when there was an overlap of interference distance, this affected the number of frames to reach the sink node due to channel access contention. This highlighted the hotspot issue, as one node would run out of energy far sooner than the others and at that point the network was in danger of becoming redundant. Even in the event that neighbouring nodes could then take on the role of hotspot when a large interference distance was in use, this may have increased network lifetime but would not avoid the ultimate conclusion, that network failure was the likely eventuality.

#### 5.4.2. Results—Average Energy Consumption

**Figure 6:**
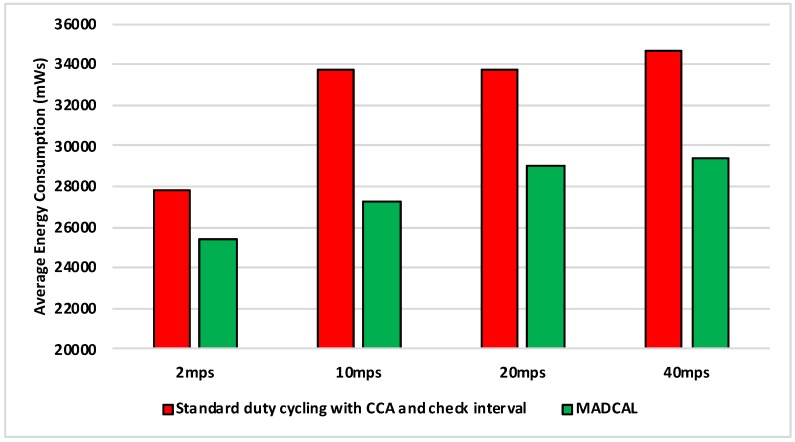
Average energy consumption (mWs), grid topology, significant nodes. Interference range 77.52 m.

**Figure 7:**
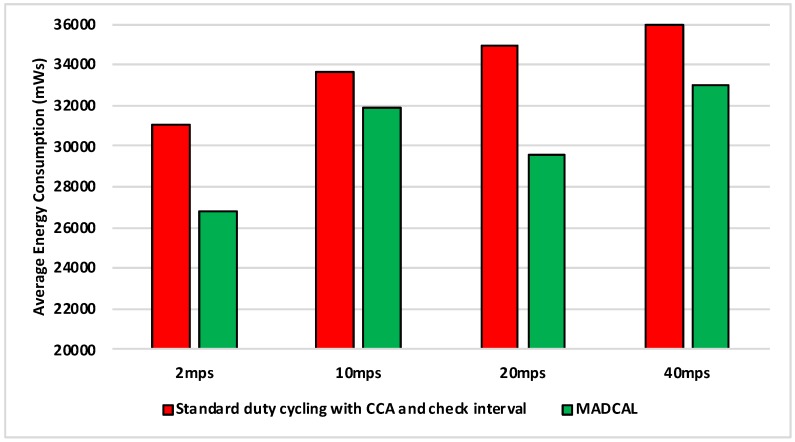
Average energy consumption (mWs), grid topology, significant nodes. Interference range 69.13 m.

**Figure 8:**
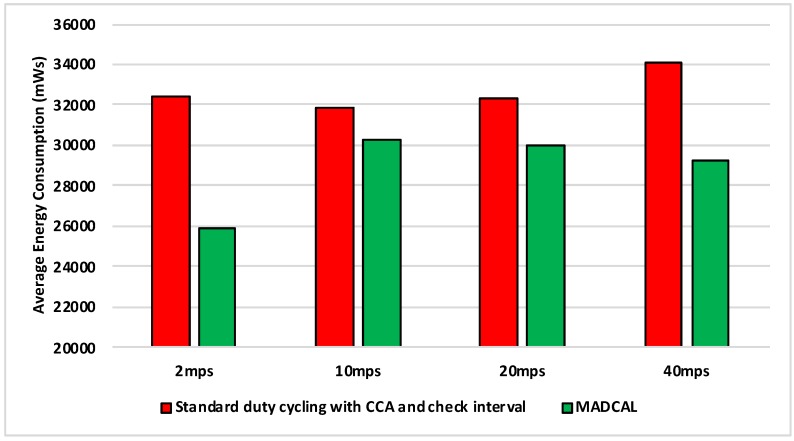
Average energy consumption (mWs), grid topology, significant nodes. Interference range 62.02 m.

**Figure 9:**
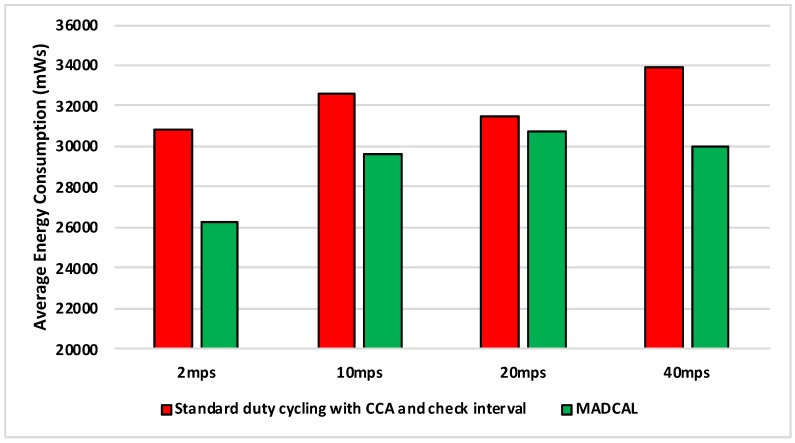
Average energy consumption (mWs), grid topology, significant nodes. Interference range 55.94 m.

Figure 6, Figure 7, Figure 8 and Figure 9 show the average energy consumption across significant nodes. This is seen as important as these are the nodes which then took on the role of hotspot, therefore reducing energy consumption in these nodes was beneficial both in terms of overall network performance and network lifetime. The comparisons shown are between the evaluation results, where the standard duty cycling with CCA and check interval was in use, and results where the MADCAL algorithm was applied, such that a dynamic threshold was created within significant nodes for communication with the MSN.

Figure 6 illustrates significant energy saving when the MADCAL algorithm was in use. The larger interference range in use here would normally result in considerable overlap of communication between significant nodes causing competition for communication with the MSN. This, subsequently, results in wasted energy consumption, with some nodes awake but unable to communicate with the sink. However, with a communication threshold established by MADCAL, although overlaps of threshold are still possible depending on node position, nodes are less likely to seek channel access at the same time. Hence there is less extraneous energy consumption.

In Figure 7 interference range is reduced to 69.13 m. Results remain a significant improvement, however, we can now observe how as interference range reduces it becomes more difficult to improve energy consumption.

There is little difference between Figure 8 from Figure 7 with benefits still to be seen in energy consumption when MADCAL is in use. One main observation is that it can be seen that it is easier to save energy when the sink is moving more slowly. In this case larger thresholds are calculated, but with the sink moving at only 2 mps, there is more time to put nodes to sleep before the sink node reaches. The counter to this is that it could result in increased delay of packet delivery.

Figure 9 is significant in that the interference range is now strained to the extent that it is only marginally greater than the distance between nodes and the greatest distance to the sink—50 m. However, despite the reduction in communication overlap when standard duty cycling is in use, the MADCAL algorithm still results in improved energy consumption, which again is most in evidence when the sink node is moving slowly.

#### 5.4.3. Results—MAC Layer Frame Delivery

**Figure 10:**
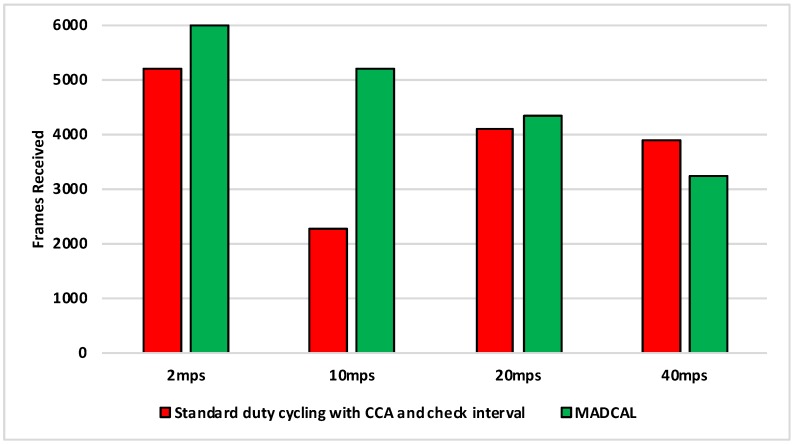
Sink frame reception, grid topology. Interference range 77.52 m.

**Figure 11:**
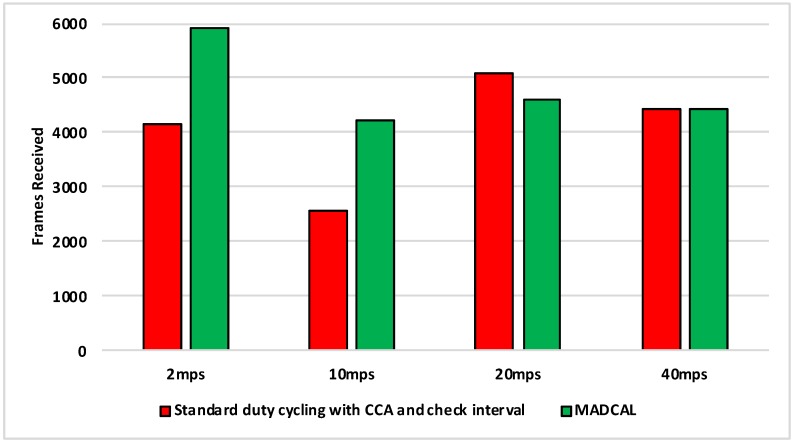
Sink frame reception, grid topology. Interference range 69.13 m.

**Figure 12:**
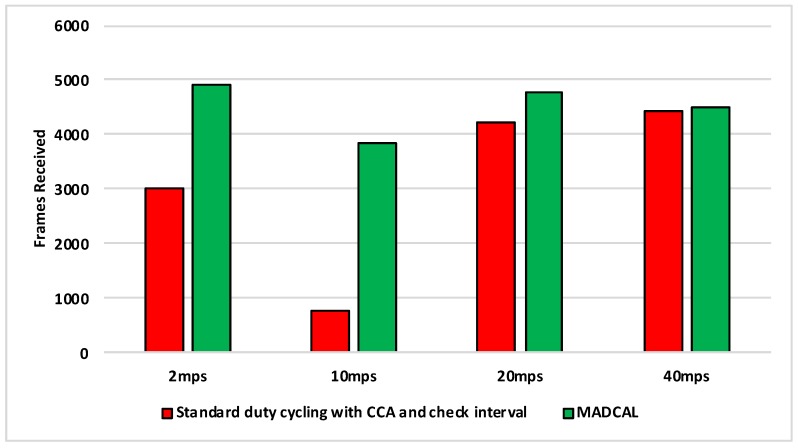
Sink frame reception, grid topology. Interference range 62.02 m.

**Figure 13:**
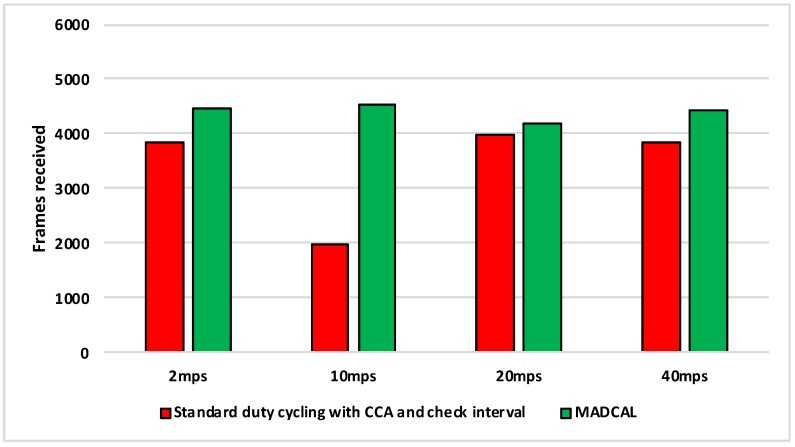
Sink frame reception, grid topology. Interference range 55.94 m.

Figure 10, Figure 11, Figure 12 and Figure 13 illustrate the number of MAC layer frames received by the sink during each simulation scenario. This is an important comparison between when MADCAL is in use and when not, as improved energy consumption would not be acceptable if detrimental to the network’s ability to function in terms of delivering packets. In observing Figure 10 it can be seen that much like energy consumption, frame reception is easier to improve upon when the sink mobility is slower. What becomes clear from our studies is that improvements are difficult at sink mobility speeds of 20 mps and higher. But frame reception which is similar, even if slightly lower, could be seen as acceptable in the event that energy consumption is significantly improved.

Figure 11 again shows the benefit of the sink moving more slowly, with frame reception the same or slightly worse for the faster speeds.

In Figure 12, again the benefits are greater when the sink moves more slowly. However, as the interference range reduces, delivery to the sink node now becomes easier at faster speeds, with MADCAL showing a slight improvement. Therefore, despite the smaller interference range, the subsequent reduction in overlap of communication, both with and without MADCAL, enables more efficient frame reception. As such, MADCAL can be seen as negating sink speed to a certain degree when considering frame delivery to the MSN.

In Figure 13 benefits once more are greater in lower speeds, but improvements can still be observed at faster speeds when MADCAL is in use. As in Figure 12, it can be observed that with MADCAL in use, frame delivery is now more consistent across all speeds.

#### 5.4.4. Summary

A significant improvement in energy consumption can be observed when MADCAL is implemented, especially so when sink mobility is slow. This would result in an increase in network lifetime, with the nodes closest to the sink path consuming less energy and therefore, living longer before battery power runs out. While improvements are significant at lower speeds, once the MSN speed increases improvements are less clear in terms of frame delivery. However, over all tests there are only two occurrences of frame delivery going down and not significantly. Therefore, any slight degradation can be offset by the benefit in energy consumption. It can also be argued that improvements in frame delivery are more difficult at faster speeds and that MADCAL is efficient in bringing the same levels of delivery to slower speeds that occur in faster speeds without the use of MADCAL. However, this highlights potential for future study with regard to optimal MSN speed. This research shows that even as speeds reach 40 mps (144 kmph), an improvement in energy consumption is possible while frame delivery remains stable.

### 5.5. Random Network Formation

#### 5.5.1. Significant Node Variance

In the previous series of results, utilising the grid topology to be found in Figure 1, no matter the interference range of the nodes, the significant nodes were unchanged throughout. With the random topology, which can be seen in Figure 2, this is not the case. As the interference range is reduced as in the previous tests, as does the number of significant nodes reduce. This a result of some now being out of range of the circular path of the MSN as the interference range decreases. Results are still given in reference to significant nodes, however, which nodes are of significance for each scenario can be seen in Table 2.

#### 5.5.2. Results—Average Energy Consumption

**Figure 14:**
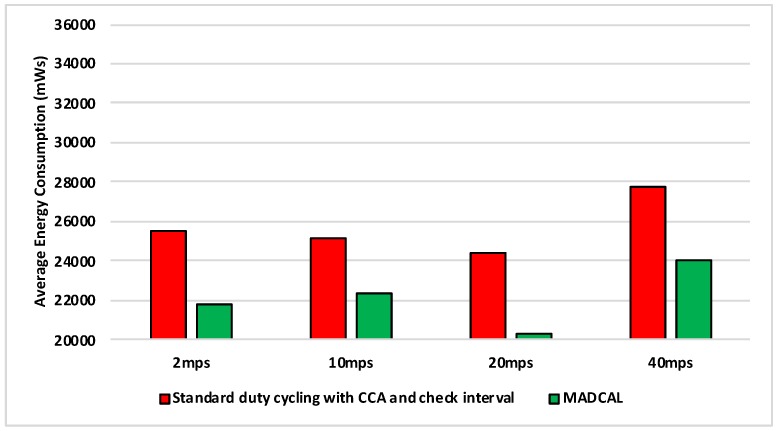
Average energy consumption (mWs), random topology, significant nodes. Interference range 77.52 m.

**Figure 15:**
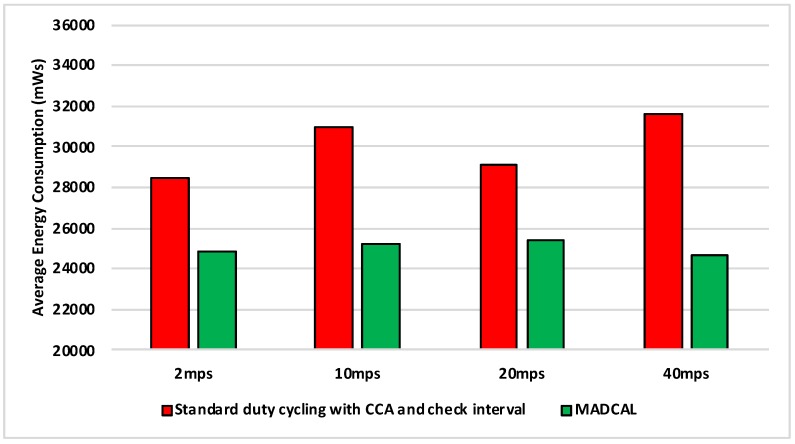
Average energy consumption (mWs), random topology, significant nodes. Interference range 69.13 m.

**Figure 16:**
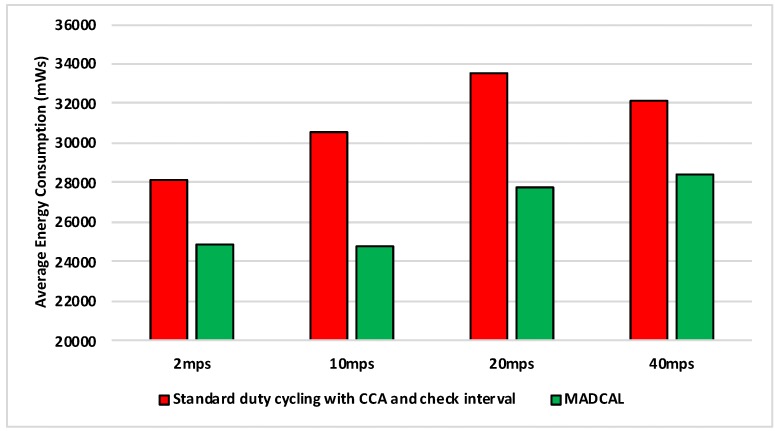
Average energy consumption (mWs), random topology, significant nodes. Interference range 62.02 m.

**Figure 17:**
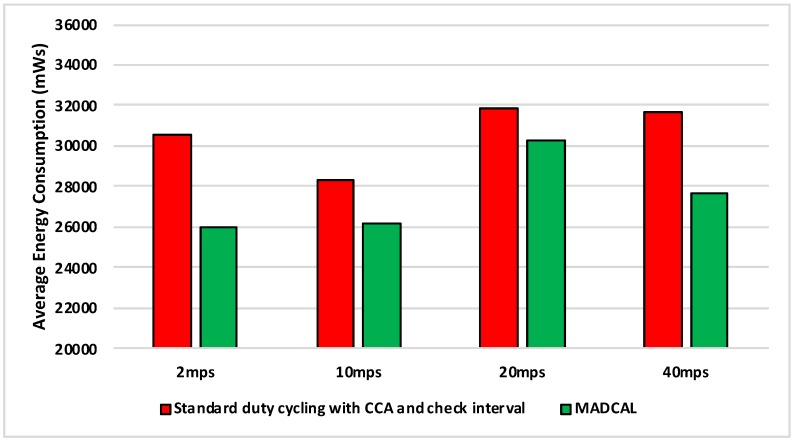
Average energy consumption (mWs), random topology, significant nodes. Interference range 55.94 m.

Figure 14, Figure 15, Figure 16 and Figure 17 show the average energy consumption across significant nodes. As with the grid topology, these nodes now take on the role of hotspot. The comparisons shown are between the evaluation results, where the standard duty cycling with CCA and check interval is in use, and results where the MADCAL algorithm is applied, such that a dynamic threshold is created within significant nodes for communication with the MSN.

Figure 14 illustrates reduced energy consumption when the MADCAL algorithm is in use. However, in comparison to the grid topology it can be seen that energy levels are lower even before MADCAL is applied. This is likely due to the large gaps between groups of significant nodes in this more random topology. Allowing for less overlap of communication over the network as a whole. This is then improved upon still by the application of MADCAL. As such we can begin to see that MADCAL is again of benefit, even in a less controlled environment such as this.

In Figure 15 interference range is reduced to 69.13 m. The results shown are now a significant improvement as the interference range decreases. However, it can be seen also that energy consumption has risen considerably in comparison to Figure 14. In this test there are now three fewer significant nodes, therefore as each takes on a greater role, more energy is consumed overall as a result.

In Figure 16 there is again benefit shown when using the MADCAL algorithm as interference range decreases again. Even more so than when a controlled, grid topology was in use.

Figure 17 is significant in that the interference range is now strained to a great extent. However, benefits can still be seen with MADCAL in use. As with the grid topology, this is less evident when the sink is moving faster. However, unlike in the equivalent test, shown in Figure 9, the benefits in energy consumption are greater overall now.

#### 5.5.3. Results—MAC Layer Frame Delivery

**Figure 18:**
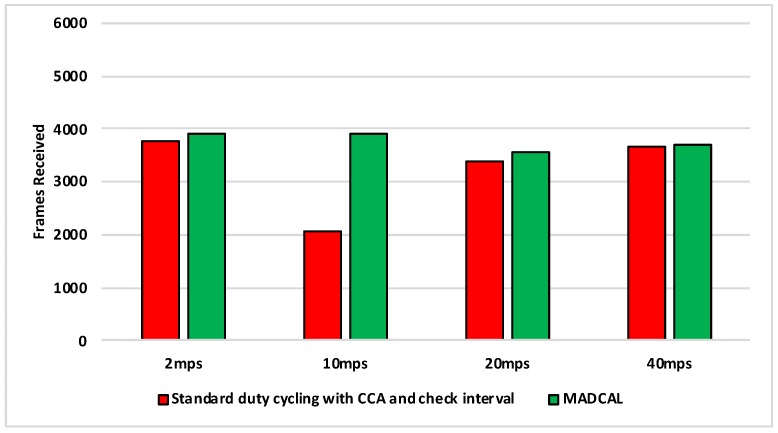
Sink frame reception, random topology. Interference range 77.52 m.

**Figure 19:**
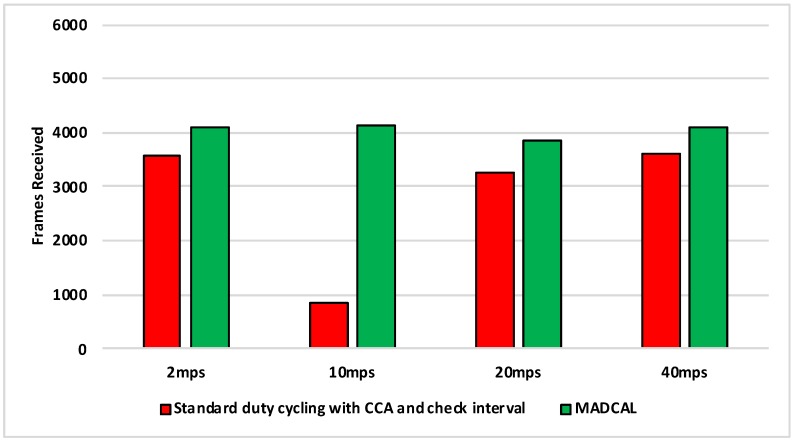
Sink frame reception, random topology. Interference range 69.13 m.

**Figure 20:**
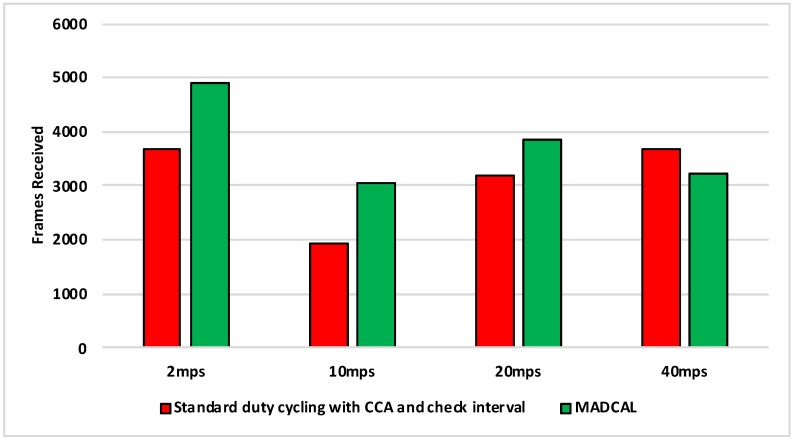
Sink frame reception, random topology. Interference range 62.02 m.

**Figure 21:**
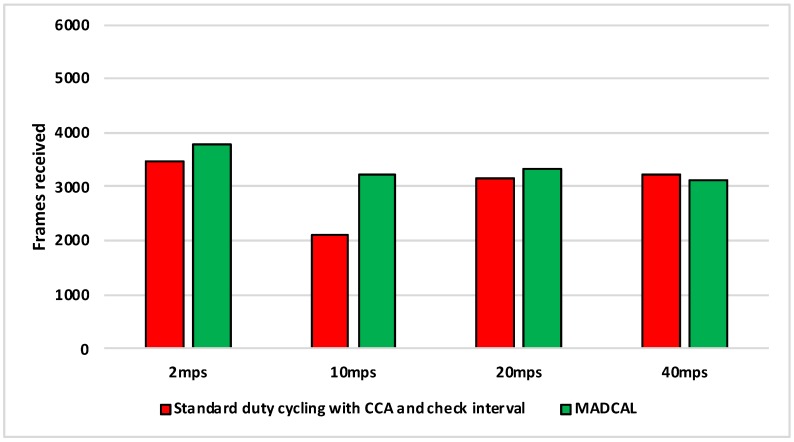
Sink frame reception, random topology. Interference range 55.94 m.

Figure 18, Figure 19, Figure 20 and Figure 21 illustrate the number of MAC layer frames received by the sink during each simulation scenario. Again this is an important comparison between when MADCAL is in use and when not, this time to see if MADCAL improves or adversely affects frame reception now a random topology is in use.

Of interest in Figure 18 is that it can be observed that MADCAL brings the frame reception into line across all different speeds in this scenario. While 10 mps was previously significantly lower than the other speeds in this regard, it was not so when MADCAL was in use. This also occurred when the topology was more controlled, but only once interference ranges became much smaller.

As with Figure 18, Figure 19 again shows that MADCAL brings the level of frame delivery to similar levels for all speeds. This time though, benefits are even more noticeable.

Now with interference range decreasing, in Figure 20 it can be seen that frame reception decreases. MADCAL, however, shows benefit in all but one scenario, where speed is at 40 mps. However, the loss is not so significant as to cause concern when the benefit in energy consumption is taken into consideration.

In Figure 21 it can again be seen that with MADCAL in use, frame delivery becomes consistent across all speeds. By now though, with the much smaller interference range, frame reception overall is lower than when the grid topology was in use. This is due to large gaps between nodes that are unable to be bridged when the interference distance is so low. A reminder of the importance of topology as a factor in the building of any network.

#### 5.5.4. Summary

As with the more controlled grid topology, a significant improvement in energy consumption is again evident when MADCAL is implemented. However, in this more random scenario there is improvement across all speeds, even as the interference range reduces. As such, compared to the controlled grid topology, the improvements in network lifetime are greater, especially so at faster sink speeds. When considering frame delivery, there are no significant improvements when using MADCAL other than at 10 mps. However, frame delivery improves slightly or stays roughly the same and is generally consistent across all speeds once MADCAL is in use. As such MADCAL can be seen as improving energy consumption considerably, whilst generally improving frame reception to the point where sink speed is mostly negated.

## 6. Conclusions and Future Work

In this paper we propose MADCAL, a dynamic and lightweight duty cycling algorithm for use in WSNs where MSNs are utilised. Results show that both where network topology is controlled and where static nodes are located randomly, when nodes are aware of their own location as well as the sink start point and speed, energy consumption can be reduced amongst significant nodes. These being the nodes which are within one-hop of the MSN path and to all intent and purpose, replace the role of nodes which previously would become hotspots where the sink node is static. This is achieved with no additional network overhead without the energy consuming exchange of messages. As such, MADCAL provides a crucial first step in the area of utilising predictable mobility patterns. In future work, certain issues still need to be addressed such as possible delay in the event of a slow moving sink, which could result in some nodes asleep for longer than may be efficient. It could be that dependant on sink speed and the size of the network, significant nodes are allowed to waken when the MSN is not within the threshold, in order that they may behave as normal sensors with data to send. We also accept that a sink mobility pattern may have to be altered at some stage. This could be due to obstacles or in the event of emergency. A such, allowances should be made for this. However, for our next study, we propose extending MADCAL further in order that the communication threshold for each node may become completely dynamic, adjusting as time passes in order to eliminate spikes in energy consumption amongst significant nodes. This would be a major step to increasing further the time until any node fails in the network.

## Figures and Tables

**Table 1:** Simulation parameters.

Test Parameters	Values
Number of Static Nodes	25
Playground Size	x = 500 m y = 500 m
Circle Radius	150 m
Sink Start Position	x = 400 m, y = 250 m
Sink Node Speed (metres per second)	2 mps, 10 mps, 20 mps, 40 mps
Simulation Time	942.47779607694 s
Interference Distance	77.52 m, 69.13 m, 62.02, 55.94 m
Number of Runs	5
Path-loss Alpha	1.85, 1.9, 1.95, 2
Carrier Frequency	2.4 GHz
Maximum Sending Power	1.0 mW
Signal Attenuation Threshold	−85 dBm
Sensitivity	−75 dBm
Transmitter Power	1.0 mW
Thermal Noise	−85 dBm
Signal to Noise Ratio Threshold	4 dB
Battery Capacity	59,400 mWs

**Table 2:** Significant nodes—random topology.

Interference Range	Significant Nodes
77.52 m	1, 2, 3, 4, 5, 6, 9, 11, 15, 16, 17, 19, 20, 21, 22, 23, 24, 25
69.13 m	1, 2, 3, 4, 5, 11, 15, 16, 17, 19, 20, 21, 24, 25
62.02 m	1, 2, 3, 4, 5, 11, 15, 16, 17, 19, 21, 24, 25
55.94 m	1, 2, 3, 4, 5, 11, 15, 16, 17, 21, 24, 25

## Abbreviations

The following abbreviations are used in this manuscript:

MAC	Media access control
WSN	Wireless sensor network
UAV	Unmanned aerial vehicle
ND	Neighbour discovery
MSN	Mobile sink node
IoT	Internet of things
MADCAL	Mobility aware duty cycling algorithm
CCA	Clear channel assessment
PDR	Packet delivery ratio
DRS	Delay-intolerant routing scheme
OLSR	Optimized link state routing protocol
mps	Metres per second
kmph	Kilometres per hour
mWs	Milliwatts per second
GPS	Global positioning system
